# Challenges in breastfeeding consultation among child health service nurses in Sweden: a qualitative study

**DOI:** 10.3389/fped.2025.1584468

**Published:** 2025-07-14

**Authors:** Pernilla Ny, Johanna Andersson, Elin Bergholtz, Ann-Christin Janlöv, Maria Ekstrand Ragnar

**Affiliations:** 1 Department of Health Sciences, Faculty of Medicine, Lund University, Lund, Sweden; 2 Health Care Centre Åhus, Åhus, Sweden; 3 Health Care Centre Näsby, Kristianstad, Sweden; 4 Department of Nursing and Integrated Health Sciences, Faculty of Health Science, Kristianstad University, Kristianstad, Sweden; 5 Department of Women’s and Children’s Health, Uppsala University, Uppsala, Sweden

**Keywords:** breastfeeding, counselling, support, child health service nurses, mothers, sustainability

## Abstract

**Background:**

In Sweden, evidence-based breastfeeding support is provided as part of the 2022–2027 Swedish Food Agency's breastfeeding strategy. Despite 98% of mothers intending to breastfeed, exclusive breastfeeding rates have dropped from 82% in 2014 to 67% in 2022. Child health service nurses offer guidance regarding breastfeeding to women and their partners. The aim of this study was to explore the challenges perceived by nurses in child health services in providing breastfeeding support to mothers in Sweden.

**Materials and methods:**

Qualitative methodology, using semi-structured interviews with 12 purposively recruited Child Health Service Nurses (CHSN) in southern Sweden. The interviews were recorded, transcribed verbatim and analysed using qualitative content analysis.

**Results:**

The analysis identified three main categories, eleven sub-categories and one overall theme: “*Taking a step back when balancing breastfeeding communication in a diverse care chain*”, which illustrates the CHSNs' struggles during consultations. It reflects communication barriers (i.e., language gaps), relational concerns (i.e., fear of harming trust), cultural factors (i.e., different perceptions of breastfeeding), and organizational hurdles (i.e., time constraints, limited access), prompting CHSNs to delicately balance support while respecting individual choices, often resulting in stepping back from evidence-based breastfeeding advice when faced with obstacles.

**Conclusion:**

The study highlights challenges in breastfeeding support among CHSNs, underscoring the need for evidence-based, person-centred care. Further education and guidance are essential to improve support and advance global health goals.

## Introduction

In Sweden, parents and soon-to-be parents are entitled to evidence-based breastfeeding support, as outlined in the Swedish Food Agency's National Breastfeeding Strategy 2022–2027 (1). Support and guidance must also align with national regulations (SOSFS 2008:33) on infant feeding, whether through breastfeeding or formula (2) and with the National Guidelines on Pregnancy, Childbirth and the Postpartum Period, developed by the National Board of Health and Welfare (3). These frameworks draw upon international recommendations, including UNICEF's guidance and the WHO's “Ten Steps to Successful Breastfeeding” (4).

With a longstanding cultural emphasis on positive breastfeeding practices, Sweden acknowledges the significant health benefits for both mothers (5) and children (6). An overwhelming 98% of Swedish mothers express the intention to breastfeed their infants (7). Despite the provision of 480 days of paid parental leave (8) and legal protections supporting breastfeeding in the workplace (9) exclusive breastfeeding rates in Sweden have declined notably over recent decades. Rates fell from 82% at 4 weeks of age in 2014 to 67% in 2022 (10), with southern Sweden showing a particularly pronounced decline. Several factors likely contribute to this multifaceted issue, including the early introduction of formula in postpartum care with a 40% usage rate in healthy newborns in the Region of Skåne (11), as well as shifting cultural norms communicated through social media and popular parenting literature (12). For example, Henriksson and Rubertsson (12) show how breastfeeding often is portrayed as a biological limitation that conflicts with ideals of equal parenting, potentially contributing to ambivalent attitudes toward breastfeeding. However, the complete picture still remains unclear.

In Sweden, child health service nurses (CHSN) are mainly responsible for providing guidance to women and their families regarding breastfeeding and overall children's health, commencing from 1 week postpartum (13). The Swedish child health service (CHS) includes a national programme for families with children 0–6 years old, with regularly scheduled encounters with a CHSN. The CHS also provides services by physicians, psychologists, speech therapists and dieticians, all free of charge.

All families are invited to participate in this voluntary programme. CHSNs have the responsibility to counsel women and their families regarding children's health in general, including breastfeeding support. CHSNs in Sweden receive basic training in breastfeeding support as part of their formal education, and some also get access to supplementary training (14). It is not a requirement that CHSNs are certified according to IBCLC standards (International Board-Certified Lactation Consultant).

The care organisation is widely embraced and esteemed by families, with nearly 100% participation in healthcare programmes for children up to 6 years of age (13). However, previous research indicates a substantial demand for updated counselling education (15) as nurses encounter challenges in addressing sensitive topics—including breastfeeding support. This can be emotionally difficult, as nurses may be reminded of past personal or professional experiences they have had to manage (16). While one recent study has explored breastfeeding counselling including the perspective of public health nurses (17), there is still a lack of research addressing how CHSNs experience and manage the complex nature of breastfeeding support, particularly within a Swedish context. Given their central role in providing breastfeeding support during the early years of a child's life, gaining insight into their perceptions and perspectives is essential for improving the quality of counselling.

The aim of this study was to explore the challenges perceived by nurses in child health services in providing breastfeeding support to mothers in Sweden.

## Method

### Study design

An exploratory qualitative research framework was adopted as outlined by Polit and Beck (2017) (18), utilising individual semi-structured interviews with CHSNs experienced in conducting breastfeeding support. Participants were selected through purposive sampling, as CHSNs hold primary responsibility for providing breastfeeding support in Sweden (13).

Inclusion criteria for participation were: (1) completion of a specialist nursing degree in either district nursing or pediatric nursing; (2) a minimum of 1 year of professional experience within child health services; and (3) regular professional involvement with breastfeeding support as part of their clinical responsibilities as Child Health Service Nurses.

The study followed the Consolidated Criteria for Reporting Qualitative Research (COREQ) guidelines (19) throughout all phases, including study design, participant recruitment, data collection, analysis, and reporting.

### Setting

The study was conducted at eleven Child Health Centres (CHC) in seven municipalities in eastern Skåne. All CHCs were affiliated with a healthcare centre, operating primarily on the healthcare centre's premises and organised under primary care. In Region Skåne, primary care has its own administration, including both private and public healthcare centres. Four healthcare centres were located in urban areas, serving populations of approximately 19,000–40,000, while seven were in rural areas, serving populations of 1,300–8,000 (20). Since the introduction of Health and Care Choice Skåne, residents can choose their healthcare centre regardless of their living area (21). Four centres were privately owned, and seven were regionally governed.

### Data collection

Data collection took place from May to October 2019. Initial contact with clinical directors at 31 healthcare centres, each with an associated CHC, was made via email, and in one case via direct approach, requesting permission to conduct the study. The emails included study information and consent forms for the clinical directors and potential informants (CHSNs). Out of the 31 clinical directors contacted, eleven did not respond, despite repeated attempts to contact them via email and phone. After three failed contact attempts, no further attempts were made. Nine directors declined participation either due to time constraints of their nurses, or due to staff shortages. Finally, clinical directors from eleven healthcare centers granted permission for the study to be conducted and shared the authors' (JA and EB) contact details with their CHSNs. Eligible CHSNs then contacted the authors directly if they wished to participate; in total 13, where one CHSN later with-drew participation.

The interviews were conducted by the second and third authors (JA and EB), alternating as moderator or observer, and took place in an undisturbed environment at the CHCs where the CHSNs worked. Two pilot interviews were conducted and later included in the study, with no alterations to the interview guide. The interviews were digitally recorded, and field notes were taken. Prior to the interviews, the informants were provided with both oral and written information explaining that participation was voluntary, they could withdraw at any time without consequence, and that all data collected would be handled confidentially. It was emphasized that no identifiable information would be included in the final report, ensuring that individual responses could not be traced back to specific participants.

A semi-structured interview guide was used for data collection, incorporating open-ended and follow-up questions to explore challenges in breastfeeding support at CHCs. The guide followed a flexible structure, allowing informants to express themselves freely. Before each interview, participants provided demographic information, including age, gender, years of professional experience (overall, in child health services, and at their current workplace), additional breastfeeding training, and a description of their work tasks. Interviews began with an open-ended question about challenges in supporting breastfeeding mothers, followed by more specific questions regarding support strategies, communication approaches, the use of the “Ten Steps to Successful Breastfeeding,” and available resources. Communication-related challenges were also explored. Follow-up prompts (e.g., “How do you feel about that?”, “Can you give an example?”) were used to encourage deeper reflection. Each interview concluded with an opportunity for participants to add further comments and consent to future contact. Interviews lasted between 16 and 30 min.

### Data analysis

The interviews were analysed inductively using qualitative content analysis, following rigorous and systematic procedures (22). Initially, three authors (JA, EB and PN) read the transcribed material several times to get a sense of the whole. Meaning units were then identified, condensed and manually divided into codes. Codes sharing similar content were consolidated and merged into manifest categories, albeit with some degree of interpretation. This process was initially conducted independently, and later collaboratively. A verification process was subsequently carried out by the primary author (PN) and the two senior authors (A-CJ and M-ER), which involved revisiting transcripts and scrutinising the categorisation by refining the organization through continuous discussion and reflection. To support analytical rigor and trustworthiness, a reflexive stance was maintained throughout the study, with regular discussions on preunderstandings, assumptions, and interpretation (22).

As the analysis progressed, discussions were held among the authors to achieve a comprehensive shared understanding of the interpretation of the findings. This led to the emergence of an overarching latent theme that encapsulates the essence of all categories identified in the results analysis.

### Ethical considerations

In accordance with Swedish legislation, this study does not require approval from the Swedish Ethical Review Authority as it does not involve patients or sensitive data. All procedures were carried out in compliance with the ethical standards established by the Committee on Publication Ethics (COPE). It is the responsibility of the authors to protect the informants from any kind of harm. The authors adhered to the ethical principles, including the requirements of information, confidentiality and utilisation (23).

## Results

In total, twelve CHSNs situated at eleven healthcare centres agreed to participate and one CHSN declined. The informants were all specialist nurses, either as primary healthcare nurses or as nurses specialised in children's health, and they all had clinical experiences with breastfeeding consultations as part of their duties. The CHSNs who agreed to participate in the study were female, aged between 32 and 61 years. Their working experience ranged from 3 to 25 years.

The analysis resulted in three main categories, eleven sub-categories and one overall theme, which are presented in Table 1 and further elaborated in the text.

**Table 1:** Results: overarching theme, categories and sub-categories.

Overarching theme: Taking a step back when balancing the breastfeeding communication in a diverse care chain	
Category	Sub-category
Professional adaptations regarding breastfeeding	Finding it difficult to capture the whole picture Experiencing language barriers as being challenging in the consultation situation Needing to adapt the counselling to the individual situation Struggling to find a balance in providing supportive counselling
Perceptions of mothers’ attitudes to breastfeeding	Lacking self-confidence in breastfeeding Having unrealistic expectations of the transition to motherhood Being negatively influenced by others Recognising the role of culture in shaping breastfeeding practices
Organisational prerequisites regarding breastfeeding	Acknowledging limited access to primary healthcare nurses Perceiving a lack of consensus regarding breastfeeding recommendations across the care chain

### Taking a step back when balancing the breastfeeding communication in a diverse care chain

The overarching theme can be summarised as the CHSNs' struggle to navigate and adapt to various perceived challenges in supporting breastfeeding. This theme was derived from their encounters with mothers during consultations, where several aspects emerged, as concluded in the categories.

In essence, the theme reflects the complexity of breastfeeding consultations, encompassing communication barriers, cultural influences, organisational challenges, and the delicate balance CHSNs must maintain in their professional role. The CHSNs endeavoured to support mothers while striving to respect individual choices, often, however, taking a step back in communicating about breastfeeding.

### Professional adaptations regarding breastfeeding

This category shed light on the practical challenges faced by CHSNs in their professional adaptations regarding breastfeeding. Understanding the mothers' perspectives was found to be difficult due to perceived barriers maintained by the mothers during consultations. Fear of miscommunication and language barriers further complicated the delivery of guidance, especially in perceived complex situations.

#### Finding it difficult to capture the whole picture

The CHSNs provided examples of challenges during professional encounters with mothers. For instance, they observed that some women seemed to maintain a facade during consultations, making it difficult for the CHSNs to understand the mothers' attitudes towards breastfeeding and consequently provide accurate information and suitable advice. The nurses also found it challenging to grasp the true feelings or thoughts of the mothers with whom they interacted. They explained that some mothers might display a certain demeanour or behaviour during consultations that did not necessarily reflect their true sentiments regarding breastfeeding. According to the CHSNs, this discrepancy could be attributed to various factors, such as reluctance to express difficulties, cultural influences, or a desire to adhere to societal expectations. This was identified as a challenge in providing correct information and appropriate advice.

But there are many [mothers] who are difficult to reach, and many who are very […] put up a facade. Because one thinks one should. And who says, “I want to breastfeed”, but they don’t really want to. (5)

Other examples of when the CHSNs found it challenging to penetrate external appearances include situations where they believed they had an agreement with the mothers about breastfeeding strategy, but on the next visit, the situation had changed without explanation. Conversely, a woman might state that breastfeeding was going well, but the child was not gaining weight. In these circumstances, the CHSNs tried to gain a deeper understanding of the situation, but often found themselves unable to reach the mother, which could result in initiating formula.

#### Experiencing language barriers as being challenging in the consultation situation

Not speaking the same language and/or using interpreters was revealed as a significant challenge in the encounters with mothers.

I sat down with a mother asking her: What is milk for you? For me, milk is breast milk, that you are breastfeeding. The mother responded: “milk is formula”. (7)

Misunderstandings often occurred even when using interpreters, and the CHSNs often felt they had no control over the communication situation. There were also mothers who preferred not to use interpreters. However, when refraining from using an interpreter, the nurses often experienced that essential nuances of the language were lost to the detriment of the consultation.

#### Needing to adapt the counselling to the individual situation

The CHSNs found it challenging to implement a person-centred approach and adapt the information to individual mothers, especially in sensitive situations, such as inadequate weight gain.

Often, children do lose weight in the first few days at the maternity ward, and maybe even when we meet them for the first time […] and it’s quite delicate what we say then. By saying “oh, the child has lost weight” or if we say beforehand that “it’s normal for the child to lose weight, and we’ll see how it has progressed today” so that it doesn’t become charged. (1)

The CHSNs stressed that breastfeeding is a technique that requires learning for both the mother and the child, and that it typically takes several weeks to establish breastfeeding routines.

Then there are challenges when breastfeeding is painful. When the mother encounters issues with her breasts, infections, wounds. That it hurts to breastfeed. (1)

Supporting mothers through this period was found particularly challenging. According to the CHSNs, their role was to offer professional advice and assistance in addressing challenges, emphasising the transient nature of difficulties. Most importantly, ensuring that women felt confident and secure proved crucial, motivating some to persist in breastfeeding.

The transition to motherhood was believed to be a great challenge for the mothers. According to the CHSNs, lack of sleep and crying babies during encounters with the nurses made it difficult for the women to listen to advice. Therefore, CHSNs tried to supplement verbal information with written materials provided during the meetings.

If babies had lost weight beyond the normal range, the CHSNs deliberated internally before advising the introduction of formula. This precautionary approach stemmed from their awareness of the potential risk that women might discontinue breastfeeding.

If the children have lost weight […] then you always reconsider. Should I say, “give some formula to boost them [the children] in the beginning”? But then, it might result in [the mothers] continuing. (6)

#### Struggling to find a balance in providing supportive counselling

The CHSNs felt they needed to tread a delicate line between encouraging the women to continue breastfeeding and respecting their desire to stop.

So, you have to weigh your words on a golden scale with a hormone-filled mom. New situation, one can be quite vulnerable. (2)

Not all CHSNs believed it was their responsibility to provide information about the benefits of breastfeeding, as they assumed the women were already aware of them.

I’m not trying to change them [the mothers]. If they’ve made up their minds, I can’t come in and say, “the WHO recommends breastfeeding until the child is two”. No, I would never do that. (4)

Almost all mothers know that breastfeeding is good, so I don’t need to tell them. That’s not my task, I think. (8)

According to the CHSNs, stating the effects of breastfeeding without being perceived as spreading propaganda was particularly difficult. They felt challenged to avoid stepping on anyone's toes while trying to help the women stay motivated to continue breastfeeding. The fear of assigning blame to women was also a concern, as they might feel ashamed if they discontinued breastfeeding. The CHSNs explained their avoidance of communicating breastfeeding benefits in their clinical practice as a way to avoid burdening the women.

One tries not to impose any personal judgment on it. Because one wants to have a good relationship […] so that they can come to me with their questions. I want that even if they choose not to breastfeed. I still want to have a good relationship because it's important in other ways. (3)

### Perceptions of mothers' attitudes towards breastfeeding

The following category and sub-categories focus on the CHSNs' perceptions of mothers' attitudes towards breastfeeding and their ability to communicate adequately about it. The CHSNs observed that mothers often lacked confidence in breastfeeding, influenced by past experiences, familial attitudes, and partner opinions. Unrealistic expectations and societal pressures also impacted mothers' decisions, making it difficult to effectively communicate the importance of breastfeeding. Cultural factors, including the influence of social media, further contributed to these challenges. Most significant was the CHSNs' fear of communicating about breastfeeding without violating the mothers' opinions.

#### Perceiving mothers to be lacking self-confidence in breastfeeding

The CHSNs perceived that if mothers had a previous negative experience with breastfeeding, other women in their family who did not breastfeed or had experienced abuse, or had prior negative contact with the CHSN, it affected their ability to breastfeed.

If they have bad experiences of breastfeeding from before or contacts with Social authorities from before, then, I think communication with breastfeeding mothers is difficult. (2)

It was also noted that some mothers believed breastfeeding should come naturally, and when they experienced difficulties, they lost faith in themselves, which was expressed as insecurity. These women were perceived to need repeated confirmation from the CHSNs.

Many [mothers] have worries, and they kind of want confirmation often that they are doing it right and ask many questions about what to do. They don’t dare to trust their own intuition […] instead, you need to constantly confirm that they are doing it right and how to do it and how often should they do it. (3)

Being admitted to the Neonatal unit after delivery, with strictly scheduled feeding routines, was found to negatively affect the self-trust of mothers, according to the informants. The CHSNs encountered challenges when assisting mothers with prior experience in the Neonatal ward who were accustomed to feeding their babies on demand. These mothers had been conditioned by the hospital to strictly adhere to breastfeeding regulations, presenting additional complexities for CHSNs in providing support for free feeding.

If they [the mothers] have had the children in the neonatal unit for a while […], then it’s like: now they [the children] should have 30 millilitres, now they should have 40 millilitres, very intense. Then I come and say “now it’s free breastfeeding”. It becomes really difficult. (2)

The CHSNs perceived that the women often lacked the ability to read their babies' signals, for example, whether the babies showed signs of hunger. The CHSNs believed that this perceived lack of confidence and knowledge often led mothers to choose formula instead of breast milk. By doing so, it gave the mothers a sense of control, according to the CHSNs. When mothers and their families returned home and the baby was stable, a new set of challenges emerged. Supporting women in breastfeeding during this uncertain period and fostering trust in themselves was considered difficult. Some CHSNs utilised breastfeeding observation to optimise care and support. This also involved simple actions, such as teaching women how to hold their babies while breastfeeding. Although weight control was occasionally used as a supportive measure, CHSNs made it clear to women that it could also cause stress. Instead, they encouraged mothers to build trust in their own instincts and observe their baby.

#### Having unrealistic expectations in the transition to parenthood

CHSNs described how (conveyed that) contemporary women express a heightened desire for personal time, in contrast to earlier generations. This, in turn, created uncertainty for the nurses in terms of how to effectively communicate and respond to this evolving need. The nurses perceived the active lifestyles led by these women as a potential hindrance to successful breastfeeding.

They [mothers] should, despite having children, fulfil themselves and find time to exercise and enjoy a latte downtown. (3)

Furthermore, they observed that mothers gave up breastfeeding too easily, as bottles and formula are readily available and may be seen as an easy solution. This is also true for those who want to avoid breastfeeding publicly for various reasons.

Many often choose to quit breastfeeding too early. If it goes a bit against them, they’d rather quit. They think it should be that simple, that everything should just work, and the smallest setback makes them choose to quit. (3)

CHSNs also found that mothers often had unrealistic expectations about breastfeeding. They believed that the first time would be just joyful and trouble-free. When these expectations were not met, they felt disappointed.

#### Being negatively influenced by others

The opinions of those in the surrounding environment regarding breastfeeding, as well as the partners' attitudes, were considered challenges that directly impacted breastfeeding. The CHSNs also observed that the use of social media presented challenges for supporting breastfeeding. They felt that it was not readily apparent to the women to accept advice from them, as it did not seem obvious to the mothers that their advice was the best.

You get so much advice; it’s difficult for them [mothers] to filter; who should one believe? And it might not be as certain that we [Child Health Services] possess the best knowledge. Before, whatever healthcare said was considered law. (2)

According to the CHSNs, the striving for equality in Swedish society might lead women to believe that breastfeeding hinders achieving an equal relationship. They noted that formula feeding allowed for the inclusion of partners, for example, by enabling partners to experience as much physical contact as mothers. The CHSNs admitted their difficulty in promoting breastfeeding while also advocating for gender equality, as they found it challenging not to pit these two aspects against each other.

People are very much into equality, which is great, but it’s a bit at the expense of breastfeeding, I think. Because there’s a lot of emphasis on the dad being involved in everything. So, I can find it a little challenging to explain how important breastfeeding actually is. (12)

The CHSNs also described how siblings could complicate breastfeeding, as their attention seeking behaviour was found to disturb the mother.

Then, it’s also a challenge as a mom if you have other children. If you have a little one running around causing mischief or if you can’t see that [sibling], then you wonder what he or she is doing now, and you’re on high alert trying to breastfeed the little one. Or if the sibling wants to come up in your lap and be involved, almost stepping on the little one. (1)

#### Recognising the role of culture in shaping breastfeeding practices

The CHSNs used the terms “cultural” or “traditional” when contemplating the behaviours of women from other countries regarding breastfeeding. The nurses categorised foreign-born women into distinct groups—those who breastfed and those who more readily opted for formula. However, all foreign-born women were characterised as being reluctant to breastfeed publicly or in the presence of men. Consequently, if a male interpreter was present, women felt inhibited from breastfeeding. Therefore, the CHSNs considered using a phone interpreter as a favourable option.

I try to use professional translation over the phone so that they [the mothers] do not need to expose their breasts. (2)

### Organisational prerequisites regarding breastfeeding

This category highlights perceived challenges within the CHSNs' own organisation and in collaboration with other entities involved in caring for breastfeeding mothers, such as maternity healthcare and postpartum care. Limited access to primary healthcare nurses at the child health service after childbirth and inconsistent breastfeeding recommendations across the care chain were suggested to create uncertainties for mothers. The organisational structure, including early discharge from postpartum wards, was explained to affect the nurses' ability to provide timely assistance.

#### Acknowledging limited access to primary healthcare nurses

The CHSNs acknowledged that women need support to maintain the breastfeeding after returning home from the delivery ward. This support could be via phone, home visits or other ways. However, it was not always easy to be available for the mothers, as issues could also arise when Child Health Services was not open. Due to complicated organisational systems, the CHSNs perceived that mothers had difficulties in reaching the nurses even during working hours. They emphasised the importance of ensuring that care and assistance are readily available and accessible to women when required.

The Child Health Centre (CHC) needs to be accessible on weekdays during working hours. (1)

#### Perceiving a lack of consensus regarding breastfeeding recommendations across the care chain

CHSNs experienced that antenatal care only provided information about the positive effects of breastfeeding, without informing mothers about potential pain and problems that might occur.

I think there should be more information about maternity care. I often hear it when they [the mothers] come here later that too little has been talked about. I feel that maternity care says that breastfeeding is great and that you should do it. There’s no discussion about these difficult things; that it can be very painful, that there can be terrible tension in the breasts, that you can become sad, and that it’s sweaty. (12)

Returning home early from the postpartum ward was considered a contributing factor to discontinuing breastfeeding, as mothers had not been given sufficient time to address difficulties and seek assistance. In some cases, breastfeeding had already ceased before the first postpartum visit (within 1 week). Introducing formula at the postpartum ward created a considerable obstacle to the promotion of breastfeeding, contributing to a sense of disillusionment among the CHSNs in carrying out their responsibilities.

It’s a dilemma for us because […] if you’ve got it [formula] in there, they [maternity ward] know what to provide, and […] they [maternity ward] can say, “on the first day, you should give 10 ml, and on the second day, 20 ml, and on the third day, 30 and 40”, and if we visit them at home after five days […] then they are up to 50 ml at each feed. Can you establish breastfeeding with that? It’s impossible. It’s like completely doomed. (8)

## Discussion

Providing support for breastfeeding is a delicate act, as shown in this Swedish context, exploring the complexity from the CHSNs' perspective. Several factors are likely to contribute to the multifaceted issue of decreasing breastfeeding rates, and there is a demand to find out more about communication regarding various aspects of breastfeeding support.

Furthermore, the postpartum period has been assessed as increasingly challenging for new parents in Sweden, as reported in 2023 (24); with hospital stays averaging 1.7–3 days, among the shortest in Europe, opportunities for early support may be limited. In addition, only 57%–75% felt satisfied and involved in their care, and merely 26% of women were satisfied with the support they received for their own health during the postpartum period (24).

The aim of this study was to explore the challenges CHSNs experience while providing breastfeeding support to mothers, resulting in the main theme of *Taking a step back when balancing breastfeeding communication in a diverse care chain*.

The category *Professional adaptations regarding breastfeeding* showed that the CHSNs faced difficulties in communicating and providing support during breastfeeding consultations. The nurses expressed challenges due to factors out of their reach, such as women not being adequately prepared earlier in the care chain, both during pregnancy and postpartum care, about the realities of breastfeeding. According to the nurses, this could affect women's expectations about handling breastfeeding obstacles, as well as their expectations about motherhood. The CHSNs expressed that their main responsibly was to maintain a positive relationship with the women and not to be experienced as intrusive. Establishing functioning breastfeeding is critical when parents first arrive home with their newborn child.

Some nurses expressed “that they did not see it as their duty to inform women about the positive effects of breastfeeding”, thereby not giving them the possibility to make an informed choice. This position is in contradiction to the ethical guidelines for nurses (25). Infants who are given formula early have less chance of successful breastfeeding due to the physiological interplay that occurs between the child's demand and the stimulation of the woman's breasts (26). Other research has shown that women need emotional support (27) to develop self-esteem in their motherhood and in their interactions with their partners (28). Some of the CHSNs included in the study expressed a genuine interest and desire to help breastfeeding mothers, while others expressed taking a step back, not seeing it as their responsibility if the women themselves were doubtful about breastfeeding.

To understand the dilemma and the complexity the CHSNs face, one might consider the perspective of person-centred care. Changing habits or introducing change (29) requires long-term, individual support where, in a functioning care chain, the care recipient becomes engaged and encouraged. Supporting women is possible when the caregiver sees women as individuals with their own will and experiences, recognising their responsibilities and capabilities (30, 31). The nine pillars of person-centred care align well with the WHO's/UNICEF's 10 steps to successful breastfeeding (4): (1) empathy, (2) respect, (3) engagement, (4) relationship, (5) communication, (6) shared decision-making, (7) holistic focus, (8) individualised focus, and (9) coordinated care.

Our results demonstrate that CHSNs focus on creating a relationship with women as a mean to share decision-making. The results indicate that if a woman is unsure about breastfeeding, the nurse should use evidence-based knowledge and communication skills to support her. Instead of backing down, the nurse should provide empathetic, person-centred care based on the woman's breastfeeding goals, enabling her to make an informed choice. The importance of being listened to can be key to effective breastfeeding communication, instead of letting habits take precedence (31).

In Sweden and elsewhere, women with higher levels of education tend to breastfeed for longer, while women with lower socio-economic status breastfeed less (4). This is an immense challenge for public health efforts in general and for breastfeeding specifically, as women and children who do not breastfeed miss out on the widely acknowledged health benefits (6, 32). This widens the already existing gap in equal care and access to care, which is the basis for equal health. Everyone has the right to knowledge that enables informed choices to benefit their own health and that of their children. This aligns with Sweden's gender equality policy goals (33) as well as the UN Convention on the Rights of the Child, which has been part of Swedish law since 2020 (Article 24, paragraph 2e) (34).

From a societal perspective, breastfeeding saves lives and contributes to a more environmentally friendly society. Therefore, working with breastfeeding support, not only at the individual level, but also at the societal level, involving decision-makers, is a key factor in improving breastfeeding outcomes (35). In Swedish child healthcare, breastfeeding is considered a quality indicator (36). This requires self-inspection to be carried out by operations managers at workplaces. Self-monitoring involves, among other things, investigating whether staff approaches and attitudes might impair the quality of care (37). This is in accordance with the second stage of the Ten Steps to Successful Breastfeeding: *Ensure that staff have sufficient knowledge, competence and skills to support breastfeeding* (4).

The nurses found it challenging to grasp the true feelings or thoughts of the mothers with whom they interacted. The CHSNs in this study explained that some mothers might display a certain demeanour or behaviour during consultations that did not necessarily reflect their true sentiments regarding breastfeeding. In many societies, traditional values dictate how mothers are expected to behave (38–40), and there is also a historical notion in western societies of “unreliable bodily functions” [(41), p. 79] such as breastfeeding, which might affect women's confidence in their ability, or rather inability to produce and feed their children. This study from Great Britain showed that women often try to find strategies to cope with the uncertainty of breastfeeding, in combination with their concerns about returning to a “normal” and “productive” life (39).

Breastfeeding is a relationship not only between the mother, child and her partner, but also with the surrounding society (41), including how breastfeeding mothers are encountered publicly (42) and how mass media affect women's attitudes and choices regarding breastfeeding (43). Instead of focusing on disadvantages in mothers' wishes, CHSNs should approach these mothers with openness and genuine interest in their breastfeeding goals, understanding what they need to achieve a functioning breastfeeding within their lifestyle and on their terms. This is in accordance with patient centred care (44). By doing so, they support and empower women participate fully in society with their children.

The recently published study (42) from Sweden, Australia and Ireland, over 10,000 women's experiences of breastfeeding in public were presented. The women experienced negative attitudes, such as sexualisation, negative comments and resistance to breastfeeding in public. For a woman with children, not being able to participate in society on equal terms with others violates the human rights goals set by the Swedish government (33) and the child's rights and needs according to the Convention on the Rights of the Child (34).

The category *Organisational prerequisites regarding breastfeeding* showed a lack of cooperation with other levels of care, such as ante- and postnatal care. The nurses observed that women received differing advice about breastfeeding throughout the care chain, leaving them unprepared and uncertain about which advice to trust. CHSNs are at the end of a long care chain (from maternity healthcare, delivery ward and the postnatal ward). Barimani and Hylander showed as early as 2012 (45) that professionals felt more confident when they knew what other professions in the care chain communicated. When it comes to sensitive matters such as breastfeeding support, caregivers play a key-role and are affected by the support provided by their colleagues earlier in the process. Sweden still lacks a linked documentation system in the perinatal care chain. CHSNs do not have access to previous documentation, which means they lack knowledge about the women's breastfeeding goals. This is highlighted in the Ten Steps to Successful Breastfeeding (4) as well as in patient centred care, which emphasises the importance of safeguarding the partnership by documenting mutually agreed goals in a health plan (44).

The nurses expressed that healthy newborn babies are often introduced to formula in the postnatal ward. According to the latest Swedish report, 40% of newborns in the Region of Skane have been given formula (11). This practice challenges the nurses and violates the International Code (46), which states that formula should only be given to children on medical ground and not offered to every child.

This study shows that even though we have Swedish and international guidelines approved by the WHO and the Social Welfare Board, available on web-based platforms for both midwives in antenatal and postnatal care (13, 47) as well as a generous paid parental leave, we still do not support breastfeeding women adequately.

Breastfeeding is a collective responsibility (32). Society's support functions, including healthcare, are jointly responsible for breastfeeding promotion. Without investment in breastfeeding support from politicians, civil society and healthcare organisations at the national level, the low rate of breastfeeding globally will continue to generate large socioeconomic costs, which will affect the health of future generations.

### Methodological considerations

To ensure the trustworthiness of the study, we applied and considered the concepts of credibility, dependability and transferability (18, 22). The study's results are based on interviews with twelve CHSNs, each lasting between 16 and 30 min. Although the interviews were relatively brief, which may have restricted opportunities to explore certain topics in greater detail, informants spoke openly and reflectively about their experiences and perceived challenges in breast feeding counselling.

While the participants formed a purposive sample based on their professional relevance, the number was determined by availability rather than ongoing assessment of data saturation. This may have limited the range of perspectives. However, the interviews were rich, and recurring themes emerged, suggesting that additional participants may not have substantially altered the results (48, 49).

Even if the interviews were conducted in 2015, we argue that the findings remain highly relevant. No major structural changes have been implemented in the organization of maternal and child health services in Sweden since the data were collected, nor have national guidelines for breastfeeding support, such as those in the National Handbook for Child Health services (13), been substantially revised. Furthermore, the decline in breastfeeding rates has continued throughout this period, underlining the persistent challenges described by the participants.

The purposive sample included CHSNs with varying years of experience, from both urban and rural CHCs, which support credibility. Furthermore, we analysed the interviews collaboratively, continuously discussing and reflecting on the textual material throughout the research process. This collaboration led to more consistent coding, categorisation, and consensus on the final theme. Adherence to COREQ strengthened the study's methodological rigor, enhanced transparency, and supported the credibility and trustworthiness of the findings.

In qualitative research, authors are viewed as co-creators of the results during the interviewing and analytical processes (18, 50) and an important factor contributing to dependability is the authors' ability to distance themselves from the material during analysis. The authors comprehensively discussed pre-understandings to avoid imposing personal values on the informants' stories. By including both junior and senior researchers, and from different professional backgrounds in the analytical process, we aimed to increase the dependability of the results, as interpretative perspectives can vary (51).

By providing a detailed account of the study's methodology, we strived for transparency throughout the research and analytical process, enabling readers to consider alternative interpretations and evaluate the transferability of the findings to other settings (22). While the findings cannot be extended to all nurses in child health services in Sweden, they offer important understanding of common themes and tensions that may resonate in similar contexts.

## Conclusions and implications

Positioned at the end of a long care chain, CHSNs described challenges in balancing women's preferences with their professional responsibility to promote breastfeeding. To address this, we recommend initiating breastfeeding communication early in antenatal care, supported by consistent, evidence-based education across all care providers. Women's individual breastfeeding goals should be clearly documented and accessible throughout the care chain to facilitate person-centred support. We also recommend structured, recurring training for CHSNs—such as an annual mandatory update with a knowledge test—to ensure shared, up-to-date competence. This requires active leadership from healthcare managers and systematic implementation of the Ten Steps to Successful Breastfeeding across services.

From a global perspective, CHS nurses play a crucial role. By offering evidence-based support to breastfeeding mothers, CHS nurses not only assist individual mothers, but also contribute to strengthening breastfeeding as a tool for achieving sustainable development. We need to be aware that there are different levels of knowledge about breastfeeding, even in settings where parents expect to find experts.

Future research should explore organizational responsibilities, timing and content of breastfeeding communication, and how to support women's individual goals within the care chain through structured, person-centered training.

## Data Availability

The datasets are not available as excerpts of raw data would potentially violate the agreement upon which participants consented.
